# Biological Functions and Prognostic Value of Ferroptosis-Related Genes in Bladder Cancer

**DOI:** 10.3389/fmolb.2021.631152

**Published:** 2021-11-17

**Authors:** Kezhen Yi, JingChong Liu, Yuan Rong, Cheng Wang, Xuan Tang, XiaoPing Zhang, Yunhe Xiong, Fubing Wang

**Affiliations:** ^1^ Department of Laboratory Medicine, Zhongnan Hospital of Wuhan University, Wuhan, China; ^2^ Department of Urology, Union Hospital, Tongji Medical College, Huazhong University of Science and Technology, Wuhan, China; ^3^ Institute of Urology, Union Hospital, Tongji Medical College, Huazhong University of Science and Technology, Wuhan, China; ^4^ Department of Hand Surgery, Union Hospital, Tongji Medical College, Huazhong, University of Science and Technology, Wuhan, China; ^5^ Urology Department, Renmin Hospital of Wuhan University, Wuhan, China; ^6^ Center for Single-Cell Omics and Tumor Liquid Biopsy, Zhongnan Hospital of Wuhan University, Wuhan, China; ^7^ Wuhan Research Center for Infectious Diseases and Cancer, Chinese Academy of Medical Sciences, Wuhan, China

**Keywords:** bladder cancer, ferroptosis, prognostic model construction, survival analysis, mRNA

## Abstract

**Background:** Every year, nearly 170,000 people die from bladder cancer worldwide. A major problem after transurethral resection of bladder tumor is that 40–80% of the tumors recur. Ferroptosis is a type of regulatory necrosis mediated by iron-catalyzed, excessive oxidation of polyunsaturated fatty acids. Increasing the sensitivity of tumor cells to ferroptosis is a potential treatment option for cancer. Establishing a diagnostic and prognostic model based on ferroptosis-related genes may provide guidance for the precise treatment of bladder cancer.

**Methods:** We downloaded mRNA data in Bladder Cancer from The Cancer Genome Atlas and analyzed differentially expressed genes based on and extract ferroptosis-related genes. We identified relevant pathways and annotate the functions of ferroptosis-related DEGs using Kyoto Encyclopedia of Genes and Genomes pathway enrichment analysis and Gene Ontology functions. On the website of Search Tool for Retrieving Interacting Genes database (STRING), we downloaded the protein-protein interactions of DEGs, which were drawn by the Cytoscape software. Then the Cox regression analysis were performed so that the prognostic value of ferroptosis-related genes and survival time are combined to identify survival- and ferroptosis-related genes and establish a prognostic formula. Survival analysis and receiver operating characteristic curvevalidation were then performed. Risk curves and nomograms were generated for both groups to predict survival. Finally, RT-qPCR was applied to analyze gene expression.

**Results:** Eight ferroptosis-related genes with prognostic value (ISCU, NFE2L2, MAFG, ZEB1, VDAC2, TXNIP, SCD, and JDP2) were identified. With clinical data, we established a prognostic model to provide promising diagnostic and prognostic information of bladder cancer based on the eight ferroptosis-related genes. RT-qPCR revealed the genes that were differentially expressed between normal and cancer tissues.

**Conclusion:** This study found that the ferroptosis-related genes is associated with bladder cancer, which may serve as new target for the treatment of bladder cancer.

## Introduction

The mortality of Bladder cancer ranks 13th among cancers (Antoni et al., 2017). To make a diagnosis and give treatment of bladder cancer, prognosis and risk assessment are important. The TNM staging system has been widely applied to predict the prognosis in cancer patients (Kluth et al., 2015). In recent years, the diagnostic or prognostic prediction model established by combining molecular markers and staging systems has improved the diagnosis and treatment ability of bladder cancer (Chen et al., 2020a; Yang et al., 2020). However, most molecular markers are derived from differential gene analysis, and the function of genes has not been further studied. Therefore, it is necessary to explore new functional biomarkers.

Ferroptosis is a new type of programmed cell death that is iron-dependent (Latunde-Dada, 2017; Wang et al., 2020b; Riegman et al., 2020). Ferroptosis is associated with many important physiological processes such as mitochondrial function, lipid metabolism and oxidative stress, so that induction of ferroptosis play a pivotal role in inhibiting the progression of tumor (Torti and Torti, 2020). There are increasing indications that ferroptosis can produce a marked effect in tumor treatment, and several studies have focused on cancer therapies that rely on ferroptosis (Conrad et al., 2020; Wu et al., 2020; Zuo et al., 2020). Wang et al. identified branched-chain amino acid aminotransferase 2 (BCAT2) as a novel inhibitor of ferroptosis using CRISPR/Cas9-based assays in hepatoma cells (Wang et al., 2020a). Li et al. (2019) constructed CaO_2_ and Fe_3_O_4_ nanoparticles and utilized cascade reaction-mediated ferroptosis to synergize with immune regulation in cancer treatment (Li and Rong, 2020). In a related bladder cancer study, Chen et al. (2020b) found that the quinazolinyl-arylurea derivatives can induce ferroptosis based on the structural modification of sorafenib. Therefore, genes associated with ferroptosis may predict the survival of patients with bladder cancer and new personalized therapeutic targets.

We collected mRNA expression data and corresponding clinical data from BLCA-TCGA and analyzed the biological functions of the ferroptosis-related DEGs. We then constructed a prognostic 8-gene signature associated with ferroptosis, which was validated by survival analysis and risk assessment. In addition, we performed biological functions analysis to investigate the potential mechanism of ferroptosis-related genes. These results will help to afford new insights for the prognosis and treatment of bladder cancer.

## Materials and Methods

### Data Source

The mRNA data and clinical data of bladder cancer patients were obtained from the Cancer Genome Atlas (TCGA) database (https://portal.gdc.cancer.gov/). Clinical features of bladder urothelial carcinoma patients (BLCA) from the TCGA database are shown in Table 1. The ferroptosis-related genes were then downloaded on the FerrDb database (http://www.zhounan.org/ferrdb)(Zhou and Bao, 2020). A list of related genes is provided in the Supplementary Material (Supplementary Table S1). Combined with the transcriptome sequencing map of bladder cancer, ferroptosis-related genes of bladder cancer were obtained. All data were publicly available online.

**TABLE 1:** Clinical features of BLCA patients (*n* = 412) from the TCGA database.

Variables	Patients, *n* (%)
Sex	
Male	108 (26.21%)
Female	304 (37.79%)
Age (year)	
≤65	162 (39.32%)
>65	250 (60.68%)
TNM stage	
I	2 (00.49%)
II	131 (31.80%)
III	141 (34.22%)
IV	136 (33.01%)
unknown	2 (00.49%)
T stage	
T0	1 (00.24%)
T1	3 (00.73%)
T2	120 (29.13%)
T3	196 (47.57%)
T4	59 (14.32%)
TX	1 (00.24%)
unknown	32 (7.77%)
N stage	
N0	239 (58.01%)
N1	47 (11.41%)
N2	76 (18.45%)
N3	8 (1.94%)
NX	36 (8.74%)
unknown	6 (1.46%)
M stage	
M0	196 (47.57%)
M1	11 (2.67%)
MX	202 (49.03%)
Unknown	3 (0.73%)
Grade	
High Grade	388 (94.017%)
Low Grade	21 (5.10%)
Unknown	3 (0.73%)

### Data Processing of Ferroptosis-Related Differentially Expressed Genes

Using the R software, we analyzed ferroptosis-related differentially expressed genes (DEGs) to identify differences between tumor and sample groups. The difference is significant when | log2Foldchange | > 1 and false discovery rate (FDR) < 0.05.

### Functional and Pathway Enrichment

Kyoto Encyclopedia of Genes and Genomes (KEGG) is a pathway-related database that systematically analyzes gene function and links genomic information and functional information. Gene Ontology (GO) is an international standardized classification system for gene functions that divides the functions of genes into three parts: cellular component (CC), molecular function (MF), and biological process (BP) (Yu et al., 2012). Bar graphs and bubbles were drawn by GO and KEGG analysis of ferroptosis-related DEGs. The difference is significant when *p* values < 0.05.

### Protein-Protein Interaction Network

We analyzed the PPI network of ferroptosis-related DEGs in STRING (https://string-db.org/). Subsequently, the PPI network was analyzed and visualized by Cytoscape 3.7.1, and the most relevant subnetwork module was obtained using Cytoscape plug-in MCODE.

### Identification of Prognostic Ferroptosis-Related Differentially Expressed Genes

We performed Kaplan-Meier survival analysis to assess the effect of ferroptosis-related genes on overall survival (OS) in bladder cancer patients. Then we conducted Cox regression. Prognostic ferroptosis-related genes (*p* < 0.05) were screened out. We also retained the ferroptosis-related DEGs if | log2Foldchange | >1 and FDR <0.05. The intersection of the two results in prognostic ferroptosis-related DEGs in BLCA.

### Construction and Analysis of Prognostic Models

We analyzed the prognostic value and survival time of ferroptosis-related DEGs using Cox regression analysis to identify ferroptosis-related genes associated with survival and generated forest maps. Univariate Cox regression analysis was conducted to identify the OS-related core genes, and genes with *p* < 0.1 were utilized for the subsequent multivariate Cox regression analysis. Thereafter, samples from TCGA were randomly divided into two groups in a 1:1 ratio to construct the best prognostic model, including a training group (*n* = 197) and a testing group (*n* = 196). We identified eight survival-related ferroptosis-related genes were by this model and obtained the correlation coefficients of each gene. $\overset{n}{\underset{i=1}{\sum }}=\text{ Exp}\left(\text{GENE n}\right)\text{ *coef}\left(\text{GENE n}\right)$ , where “Exp” represents the gene expression, and “coef” represents the coefficient score from multivariate Cox analysis. The risk score of each patient was calculated based on this model. Then, we analyzed the survival analysis, receiver operating characteristic curve (ROC) and risk curves of each group. Furthermore, by univariate and multivariate analysis, a nomogram was generated to provide patient survival information.

### Preparation of Bladder Cancer Clinical Tissue Samples and Adjacent Tissue Samples

Throughout the study, we obtained informed consent from each patient for the collection and analysis of tissue samples, which was approved by the Union Hospital, Tongji Medical College, and Huazhong University of Science and Technology ethics review boards. We immediately frozen and stored the tissue at −80°C after extracting it.

### Total Ribo Nucleic Acid Extraction, Reverse Transcription, and Real-Time Quantitative-Polymerace Chain Reaction Analysis

We extracted total RNA from tissue samples using Trizol reagent and used the nanodrop2000 to assess RNA integrity. We used reverse transcription reagents to react with extracted RNA to produce cDNA complementary to the single strand of RNA, which was then quantified in real time using the SYBR Green PCR kit. Each gene’s cycle threshold (CT) was recorded. The relative mean value of each gene’s expression was calculated, and the 2^-∆∆CT^ method was used to determine whether the differences between groups were statistically significant, and the mean value was used as the final experimental result for replicate wells. All procedures were carried out in accordance with the manufacturer’s instructions.

### Statistical Analysis

The predictive significance of ferroptosis associated genes and other clinical characteristics was determined by using univariate and multivariate Cox regression. The effect of this model was assessed by using Kaplan Meier curves and the log rank test method to validate the difference in survival time between different risk groups based on the ferroptosis prognostic model. The effectiveness of this ferroptosis prognostic model to predict the prognosis of bladder cancer patients was assessed using a receiver operating characteristic curve (ROC). *p* values less than 0.05 were deemed statistically significant in two-sided statistical testing. The graphing software graphpad prism version 8.0.1 and the R language version 4.0.2 were used in this investigation. The R packages mainly used in this paper include limma, pheatmap, survival, iGraph, reshape2, glmnet, clusterprofiler, org. hs.eg.db, enrichplot, ggplot2.

## Results

### Identification of Differentially Expressed Ferroptosis-Related Genes

**FIGURE 1:**
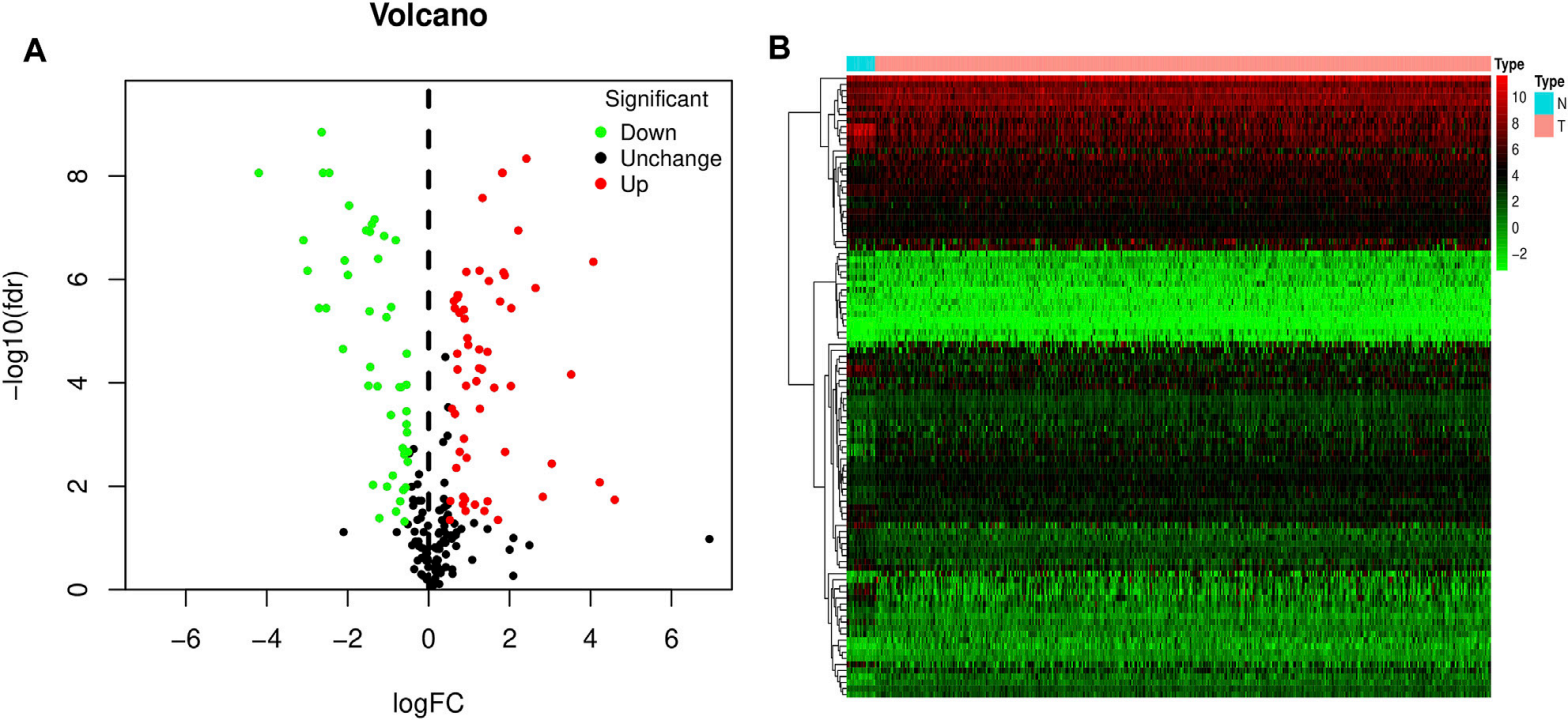
Volcano and heat map of differentially expressed ferroptosis-related genes in bladder cancer. **(A)** Volcano map. **(B)** Heat map. Red dots represent upregulated genes, and green dots represent downregulated genes.

The mRNA expression levels of all ferroptosis-related genes were compared between 19 control bladder tissues and 412 bladder cancer tissues in the TCGA-BLCA dataset. 103 ferroptosis-related DEGs were obtained, including 57 upregulated ferroptosis-related genes and 46 downregulated ferroptosis-related genes, and plotted volcano and heat maps (Figure 1).

### Functional Enrichment Analyses of Differentially Expressed Ferroptosis-Related Genes

**FIGURE 2:**
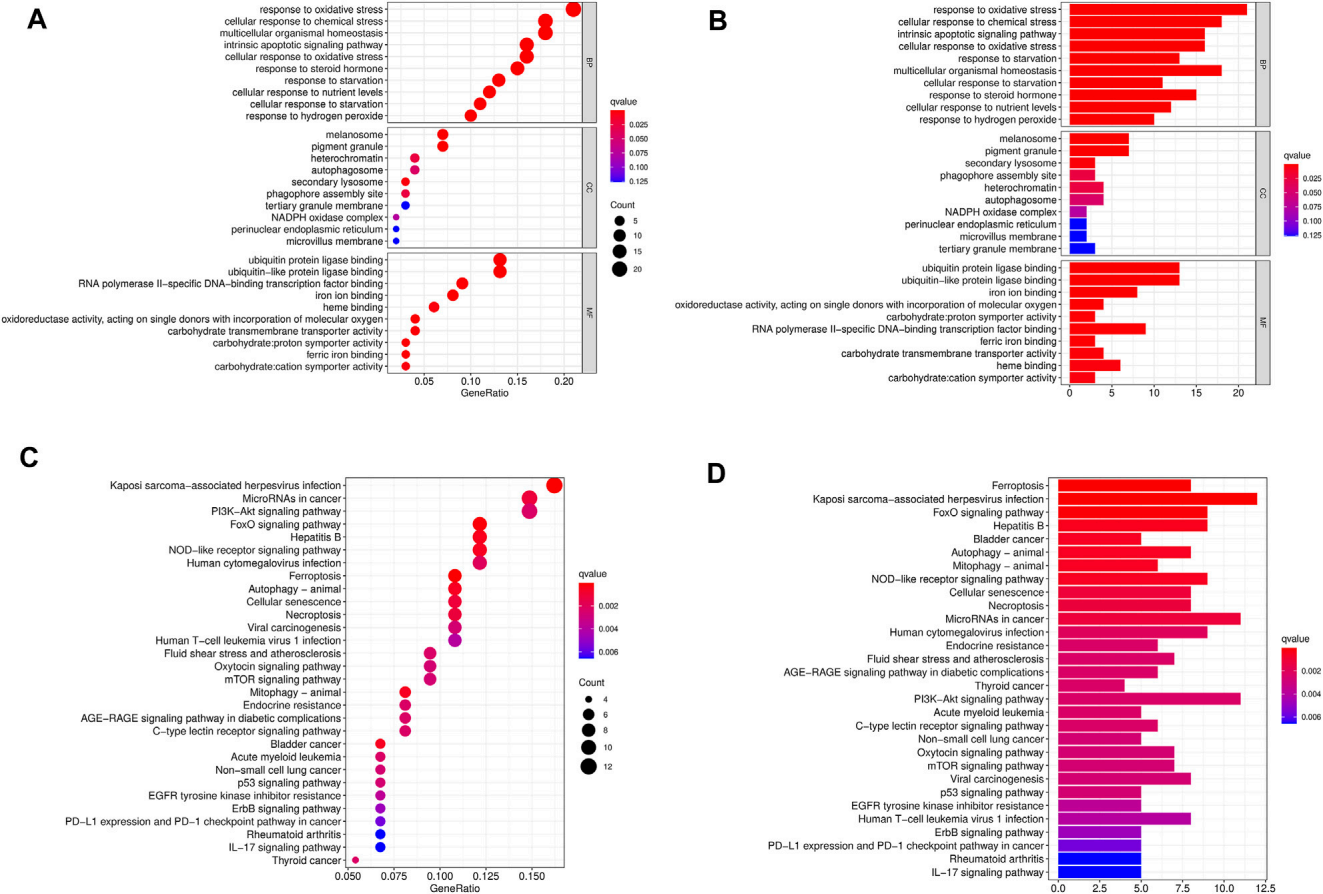
GO and KEGG enrichment pathways of differentially expressed ferroptosis-related genes. The dot graph **(A)** and bar graph **(B)** of GO and the dot graph **(C)** and bar graph **(D)** of KEGG are shown. The size of the dot indicates the count, and the color indicates the Q value.

To study the biological functions of 103 differentially expressed ferroptosis-related genes, GO function and KEGG pathway enrichment were performed, and bar plots and bubbles were plotted. As shown in Figure 2, the top 10 most relevant genes were selected. GO results indicate that differentially expressed ferroptosis-related genes are involved in some important biological processes, such as response to stimuli, maintenance of cell homeostasis, cell autophagy, apoptosis, and a variety of iron-related functions. KEGG pathway enrichment analysis indicated that 103 genes were mainly involved in PD-L1/PD-1 pathway in cancer, multiple signaling pathways related to cell differentiation, tumorigenesis and development, and autophagy-related pathways.

### Protein-Protein Interaction Network Construction

**FIGURE 3:**
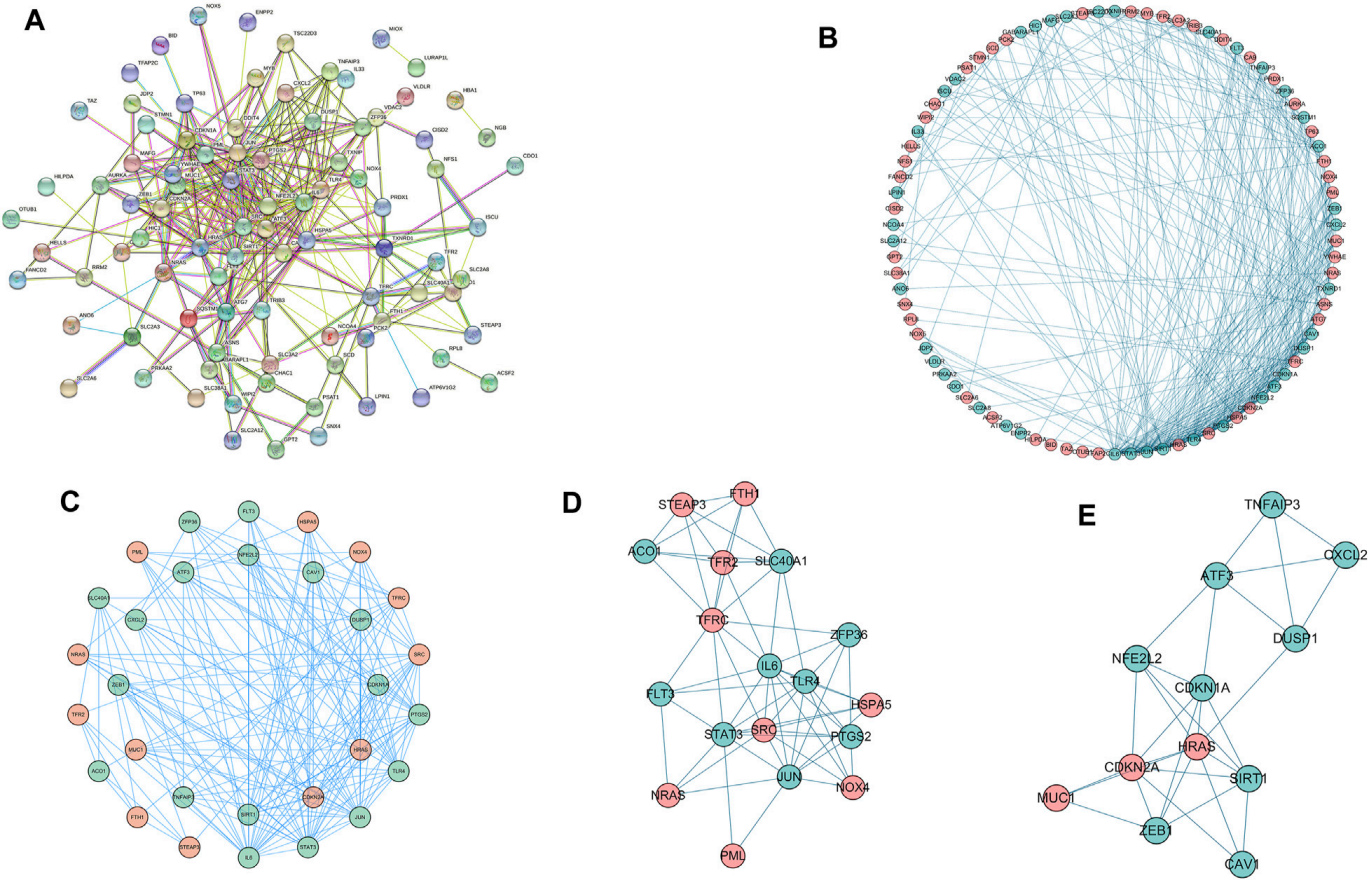
Differentially expressed ferroptosis-related genes were used to construct protein-protein interaction networks and subnetworks. **(A)** PPI interaction network map obtained from the STRING database. **(B)** Genes of the interacting PPI network visualized by Cytoscape. Red nodes represent upregulated genes, while blue nodes represent downregulated genes. **(C)** Two most significant MCODE modules visualization. **(D,E)** Two most significant MCODE components form the PPI network.

We used STRING tools to predict the protein interactions among the differentially expressed ferroptosis-related genes. We found 101 nodes and 348 edges in the PPI network. Low degree values were associated with smaller node sizes, while lower co-expression values were associated with smaller edge sizes. Thereafter, a network diagram of 102 genes was drawn using the Cytoscape software, as shown in Figure 3A. In addition, two key subnets were extracted and visualized using the MCODE plug-in, as shown in Figure 3B. Two most significant MCODE modules visualization and two most significant MCODE components form the PPI network as shown in Figures 3C-E. The results of KEGG pathway enrichment and GO function on the genes of the two subnetworks were shown in Table 2 and Table 3.

**TABLE 2:** The GO function enrichment analysis of the two most significant MCODE components.

Ontology	ID	Description	P value	Adjusted P Value	Count
Subnetwork 1					
BP	GO:0055072	iron ion homeostasis	9.84E-11	1.59E-07	6
BP	GO:0055076	transition metal ion homeostasis	2.02E-09	1.63E-06	6
BP	GO:0006879	cellular iron ion homeostasis	3.08E-09	1.66E-06	5
CC	GO:0005788	endoplasmic reticulum lumen	0.000152	0.015066	4
CC	GO:0010008	endosome membrane	0.000803	0.039742	4
MF	GO:0035258	steroid hormone receptor binding	0.000105	0.003937	3
MF	GO:0035259	glucocorticoid receptor binding	0.000116	0.003937	2
MF	GO:0061629	RNA polymerase II-specific DNA-binding transcription factor binding	0.000151	0.003937	4
Subnetwork 2					
BP	GO:0071901	negative regulation of protein serine/threonine kinase activity	1.76E-10	2.35E-07	6
BP	GO:0001933	negative regulation of protein phosphorylation	2.31E-09	1.41E-06	7
BP	GO:0006469	negative regulation of protein kinase activity	3.24E-09	1.41E-06	6
CC	GO:0000792	heterochromatin	0.000944	0.038697	2
MF	GO:0004861	cyclin-dependent protein serine/threonine kinase inhibitor activity	2.77E-05	0.002883	2
MF	GO:0051019	mitogen-activated protein kinase binding	0.000136	0.00706	2
MF	GO:0030291	protein serine/threonine kinase inhibitor activity	0.000207	0.007167	2

**TABLE 3:** The KEGG function enrichment analysis of the two most significant MCODE components.

ID	Description	P value	Adjusted P Value	Count
Subnetwork 1				
hsa05167	Kaposi sarcoma-associated herpesvirus infection	6.34E-08	9.9E-06	7
hsa05161	Hepatitis B	6.12E-07	4.78E-05	6
hsa04216	Ferroptosis	1.3E-06	5.49E-05	4
hsa04933	AGE-RAGE signaling pathway in diabetic complications	1.45E-06	5.49E-05	5
hsa04625	C-type lectin receptor signaling pathway	1.76E-06	5.49E-05	5
Subnetwork 2				
hsa05219	Bladder cancer	1.42E-05	0.000968	3
hsa05206	MicroRNAs in cancer	1.74E-05	0.000968	5
hsa04218	Cellular senescence	2.57E-05	0.000968	4

### Construction and Analysis of Prognostic Signature

**FIGURE 4:**
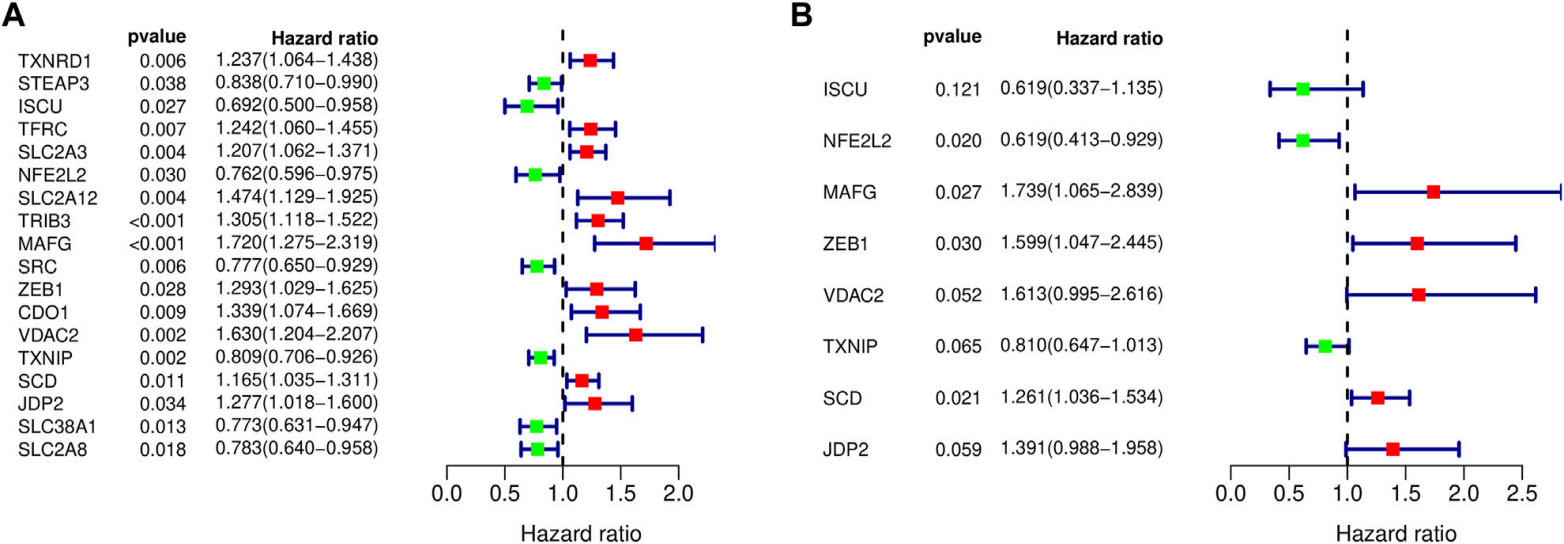
Forest maps of differentially expressed ferroptosis-related genes. **(A)** Eighteen prognostically related ferroptosis-related genes are shown in the forest map, with red denoting high-risk genes and green denoting low-risk genes. **(B)** Eight ferroptosis-related genes were obtained by constructing the prognostic model shown in the forest map.

103 differentially expressed ferroptosis-related genes interacting with survival time were analyzed to identify the prognostic value of by Cox regression; 18 survival-related genes were obtained, and forest maps were generated (Figure 4A). The samples from TCGA were randomly divided into a training set (*n* = 197) and test set (*n* = 196) according to a 1:1 ratio. Eight survival-related genes were screened out through the training set and are used to construct the best prognostic signature, and the correlation coefficients for each gene were obtained. These eight genes were: iron-sulfur cluster assembly enzymes (*ISCU*), nuclear factor, erythroid 2 like 2 (*NFE2L2*), MAF bZIP transcription factor G (*MAFG*), zinc finger E-box-binding protein 1 (*ZEB1*), voltage-dependent anion channel 2 (*VDAC2*), thioredoxin-interacting protein (*TXNIP*), stearoyl-CoA desaturase (*SCD*), and Jun dimerization protein 2 (*JDP2*) (Figure 4B). Among them, *ISCU, NFE2L2*, and *TXNIP* are classified as low-risk genes, while *ISCU, MAFG, ZEB1, VDAC2, SCD, JDP2* are classified as high-risk genes. In addition, details of the prognostic signature are listed in Table 4.

**TABLE 4:** Detailed information on ferroptosis-related genes for constructing the prognostic signature.

Id	ENSG_ID	Chromosome	Coef	HR
ISCU	ENSG00000136003	12q23.3	−0.48	0.619 (0.337−1.135)
NFE2L2	ENSG00000116044	2q31.2	−0.48	0.619 (0.413−0.929)
MAFG	ENSG00000197063	17q25.3	0.55	1.739 (1.065−2.839)
ZEB1	ENSG00000148516	10p11.22	0.47	1.599 (1.047−2.445)
VDAC2	ENSG00000165637	10q22.2	0.48	1.613 (0.995−2.616)
TXNIP	ENSG00000265972	1q21.1	−0.21	0.810 (0.647−1.013)
SCD	ENSG00000099194	10q24.31	0.23	1.261 (1.036−1.534)
JDP2	ENSG00000140044	14q24.3	0.33	1.391 (0.988−1.958)

**FIGURE 5:**
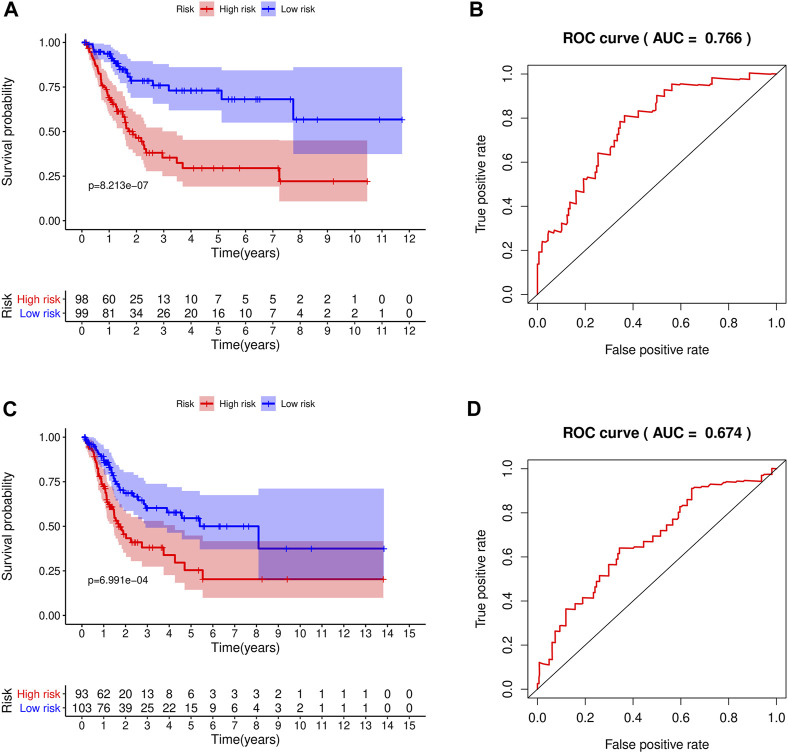
The constructed prognostic model was used for survival analysis of the training set and test set, and ROC was analyzed. **(A)** Survival analysis curve of the training set; red indicates patients in the high-risk group, blue denotes patients in the low-risk group. **(B)** ROC curve of the training set. **(C)** Survival analysis curve of the test set. **(D)** ROC curve of the test set.

**FIGURE 6:**
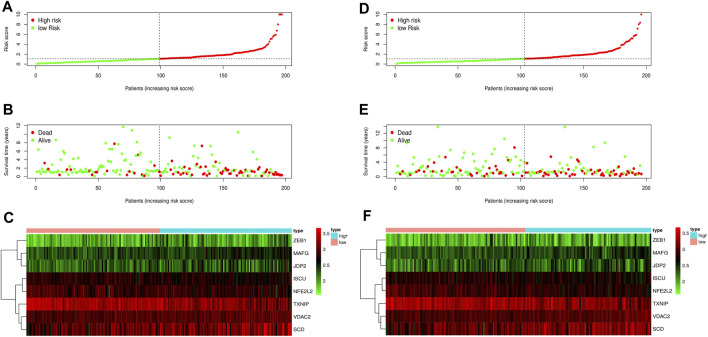
Risk curve of the training set and test set. **(A)** Risk score distribution of the training set. **(B)** Survival state distribution of training set. **(C)** Heat maps of eight ferroptosis-related genes in the training set. **(D)** Distribution of risk scores in the test set. **(E)** Survival state distribution of the test set. **(F)** Heat maps of eight ferroptosis-related genes in the test set.

We obtained a risk score for each patient and obtained the median of the risk scores for all patients according to the risk formula. The risk formula is $\begin{array}{l} Risk=Exp\left(ISCU\right)\times -0.48+Exp\left(NFE2L2\right)\times -0.48+Exp\left(MAFG\right)\times 0.55+Exp\left(ZEB1\right)\times 0.47+\\ Exp\left(VDAC2\right)\times 0.48+Exp\left(TXNIP\right)\times -0.21+Exp\left(SCD\right)\times 0.23+Exp\left(JDP2\right)\times 0.33 \end{array}$ We classified patients below the median as a low-risk group and those above the median as a high-risk group. The patients were divided into high-risk and low-risk groups in the training and test groups, respectively, according to the risk score (Figure 5A). In the training group, there were 98 patients in the high-risk group and 99 patients in the low-risk group. In the test set, there were 93 patients in the high-risk group and 103 patients in the low-risk group. As shown in the results, patients with high-risk scores had shorter survival times than those with low-risk scores. The ROC curve of survival prediction showed satisfactory performance (Figure 5B). The AUC (Area Under Curve) value in the training set was 0.766, and that in the test set was 0.674 (Figure 6). The closer the AUC value is to 1.0, the more authentic the detection method is. The same analysis was also performed in the test group with consistent results (Figure 5C,D).

**FIGURE 7:**
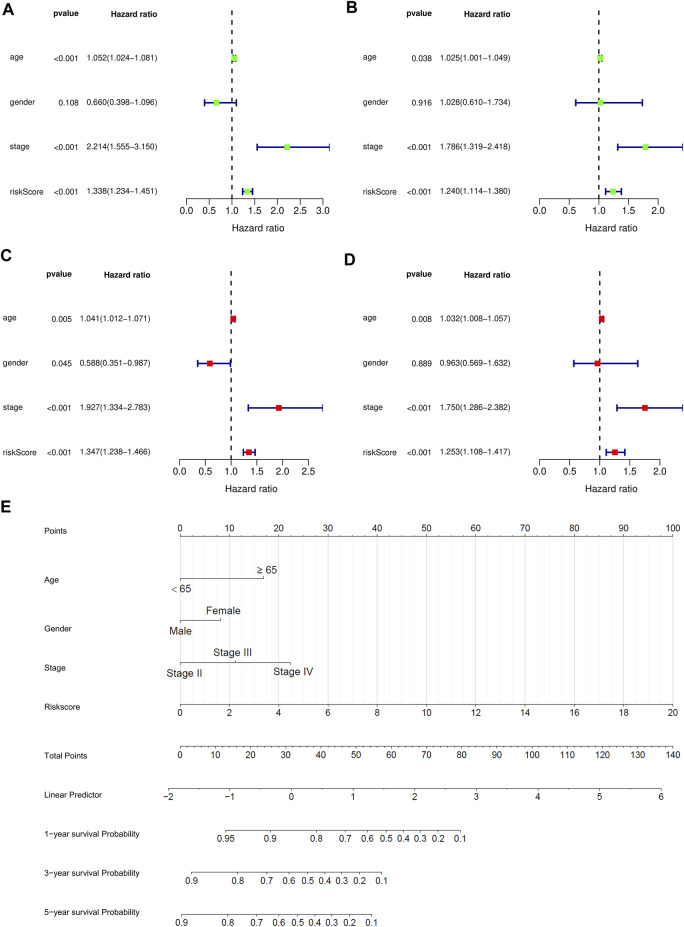
Independent prognostic analysis and 1 -, 2 -, and 3-years survival prediction were performed for bladder cancer patients in the training and testing sets. **(A)** Univariate prognostic analysis of training set. **(B)** Multivariate prognostic analysis of training set. **(C)** Univariate prognostic analysis of test set. **(D)** Multivariate prognostic analysis of test set. **(E)** The nomogram was used to predict 1-year, 2-years, and 3-years survival rates of bladder cancer patients in the training set.

We performed univariate and multivariate independent prognostic analysis. Age, tumor stage, and the risk score could be used as independent prognostic factors for survival of bladder cancer patients in the training and validation sets (*p* < 0.05) in the univariate independent prognostic analysis. Sex, age, tumor stage, and the risk score were independent prognostic factors of bladder cancer (*p* < 0.05) in the multivariate independent prognostic analysis. For the test set, age was not found to be a prognostic factor (*p* = 0.889) (Figures 7A-D). All the prognostic factors were included in the nomograms (Figure 7E).

Finally, the nomogram of these eight prognostic genes was drawn in the training set. The RNA expression of eight genes was used as a parameter to draw the dotted lines in the nomogram and the total score can predict the survival rates.

### Validation of Ferroptosis-Related Genes

**FIGURE 8:**
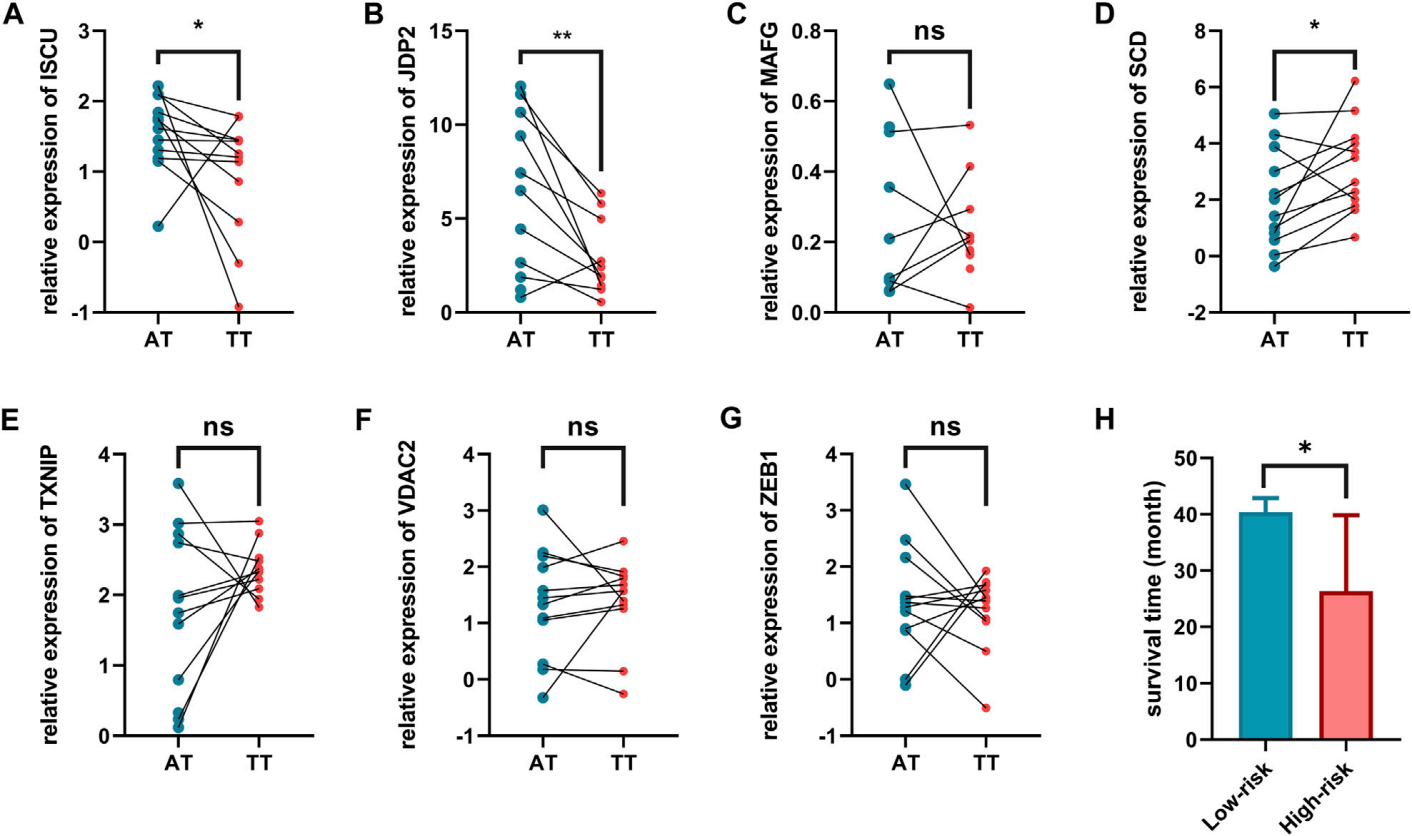
Validation of expression levels of eight ferroptosis related genes in tumor tissues. and adjacent normal tissues. **(A)** ISCU, **(B)** JDP2, **(C)** MAFG, **(D)** SCD, **(E)** TXNIP, **(F)** VDAC2, **(G)** ZEB1, **(H)** Comparison of survival time among patients with different risk characteristics. AT, adjacent normal tissues. TT, tumor tissues.

We further validated the expression of these eight genes in 12 pairs of clinical sample tissues (Figure 8). The results showed that the expression levels of ISCU and JDP2 were lower in tumor samples and higher in paracancerous tissues. In clinical sample tissues, the expression of SCD was exactly the opposite. Although the *p*-value was not less than 0.05, we found that MAFG with VDAC2 presented higher expression in paracancerous tissues than in cancer tissues, and TXNIP presented lower expression in paracancerous tissues than in cancer tissues. The low abundance of nfe2l2 expression in tissues precludes its detection by qPCR. Because the number of clinical samples was small and the plotting of survival curves was difficult, we divided the patients into high-risk and low-risk patients by the risk model derived in the previous study and found a statistical difference in survival time between these two groups. Low-risk patients had a longer survival time than high-risk patients (Figure 8H).

## Discussion

Bladder cancer has a certain recurrence rate after operation. Therefore, it is meaningful to find new diagnostic biomarkers or therapeutic targets for bladder cancer. Ferroptosis is associated with many physiological and pathological processes, such as tumor suppression, neuronal degeneration, antiviral immune response, and ischemia-reperfusion injury. From the perspective of cancer therapy, we hope to eliminate cancer cells by promoting ferroptosis or inhibiting ferroptosis in healthy cells. However, the specific mechanism of ferroptosis remains to be investigated in bladder cancer.

We integrated mRNA data from TCGA database and identified 103 ferroptosis-related DEGs, including 57 upregulated and 46 downregulated ferroptosis-related genes. In addition, through functional analysis, we found that ferroptosis-related DEGs were involved in apoptosis and immune checkpoint pathways. We then established a prognostic model based on eight ferroptosis-related genes to predict the accurate survival for patients with bladder cancer based on these results. By combining important clinical data and risk scores, a nomogram was constructed to predict survival rates.

Ferroptosis-related genes in this study included *ISCU, NFE2L2, MAFG, ZEB1, VDAC2, TXNIP, SCD*, and *JDP2*. These genes have been studied in metabolic processes or tumor development. ISCU is an important scaffold protein and can assemble iron and sulfur into iron-sulfur clusters These clusters participate in the maturation of [2Fe-2S] and [4Fe-4S] proteins in the mitochondria and cytoplasm and the regulation of iron metabolism (Duan et al., 2009). As an important redox center, iron-sulfur clusters participated in many physiological functions such especially in metabolism (Xu and Møller, 2011). *NFE2L2* is transcription factor, which regulate genes that contain antioxidant response elements in their promoters. NFE2L2 plays a vital role in the treatment of neurodegenerative diseases and regulation of ferroptosis (Liu et al., 2020; Song and Long, 2020). MAFG is a small MAF protein (Liu et al., 2018). MAFG-mediated cisplatin resistance lies in lower reactive oxygen species production; therefore, MAFG may be a potential therapeutic target (Vera-Puente et al., 2018). ZEB1 is a transcriptional repressor. Studies have shown that ZEB1 is abnormally expressed in many liver diseases, including hepatocellular carcinoma (Li et al., 2019). Studies have found that ZEB1 overexpression increases sensitivity to ferroptosis (Lee et al., 2020). *VDAC2* is a member of the voltage-dependent anion channel pore-forming protein family, which is thought to play an important role in the metabolite diffusion process on the outer mitochondrial membrane. Moreover, it can participate in the mitochondrial apoptotic pathway by regulating the activity of BCL2-antagonist/killer 1 protein. *TXNIP* is a thioredoxin-binding protein, which participates in cellular redox signaling and plays an important role in oxidative stress. TXNIP has been found to be involved in the autophagy and ferroptosis of a class of protein disulfide isomerase inhibitors in the treatment of glioblastoma (Kyani et al., 2018). *SCD* encodes enzymes involved in fatty acid biosynthesis. In one study, *SCD* expression in bladder cancer patients was found to be significantly associated with poor prognosis. Loss-of-function experiments further revealed that inhibiting SCD expression can reduce cell proliferation and invasion (Piao et al., 2019). JDP2, a member of the bZIP transcription factor family and acts as a broad AP-1 inhibitory protein. JDP2 plays an important role in inhibiting AP-1-driven biological processes, such as cell cycle regulation, cell differentiation, apoptosis, and tumorigenesis (Chen et al., 2017). In hepatocellular carcinoma, miR-501 can regulate JDP2 to promote tumor cell development (Yu et al., 2019). In conclusion, these eight genes are mainly involved in iron metabolism, lipid metabolism, and amino acid metabolism.

In general, we investigated the biological function and prognostic value of ferroptosis-related genes and established a new prognostic model based that are closely related to the overall survival of bladder cancer patients.

The main limitation of this study is the small number of clinical samples used for validation, which requires further investigation. And we need to experimentally validate the hypothesis. Also, more validation research is needed to explore the mechanism of ferroptosis-related genes in bladder cancer. In spite of all its limitations, our results suggest that prognostic markers based on ferroptosis-related genes may be a reliable predictive tool for the prognosis of bladder cancer patients.

## Data Availability

The data analyzed in this study were obtained from publicly available datasets. The mRNA data and clinical can be found here: The Cancer Genome Atlas (https://portal.gdc.cancer.gov/). The ferroptosis-related genes are downloaded from the FerrDb database (http://www.zhounan.org/ferrdb).
