# Supplementary material for: Biological Functions and Prognostic Value of Ferroptosis-Related Genes in Bladder Cancer
Source: Front Mol Biosci. 2021 Nov 17;8:631152. doi: 10.3389/fmolb.2021.631152 (PMC8635965; doi:10.3389/fmolb.2021.631152)
Supplement: Supplementary file 2 [file Table1.DOCX]

Table S1 The ferroptosis-related genes in FerrDb database

| gene | conMean | treatMean | logFC | pValue | fdr |
| --- | --- | --- | --- | --- | --- |
| ACO1 | 10.09363 | 6.544184 | -0.62516 | 0.005649 | 0.011821 |
| ACSF2 | 8.58309 | 15.45098 | 0.848129 | 0.011991 | 0.022332 |
| ALOXE3 | 0.145389 | 0.53836 | 1.888657 | 0.000866 | 0.002173 |
| ANGPTL7 | 2.335506 | 0.127292 | -4.19752 | 2.66E-10 | 8.74E-09 |
| ANO6 | 24.69499 | 13.37227 | -0.88497 | 0.002728 | 0.006222 |
| ARRDC3 | 24.19161 | 15.52479 | -0.63993 | 0.000695 | 0.001833 |
| ASNS | 5.036099 | 9.214754 | 0.871639 | 0.000444 | 0.001202 |
| ATF3 | 108.5405 | 19.78328 | -2.45588 | 2.15E-10 | 8.72E-09 |
| ATG7 | 2.684304 | 3.997023 | 0.574377 | 0.000105 | 0.000317 |
| ATP6V1G2 | 0.502439 | 0.331127 | -0.60156 | 0.000994 | 0.002432 |
| AURKA | 2.264394 | 12.07689 | 2.415053 | 4.56E-11 | 4.63E-09 |
| BID | 5.819311 | 14.64939 | 1.33192 | 1.05E-09 | 2.67E-08 |
| CA9 | 1.753627 | 20.13247 | 3.52111 | 1.90E-05 | 6.90E-05 |
| CAPG | 54.83054 | 136.6204 | 1.317121 | 1.48E-05 | 5.52E-05 |
| CAV1 | 111.1226 | 46.335 | -1.26198 | 3.57E-05 | 0.000117 |
| CDKN1A | 160.5669 | 84.13698 | -0.93236 | 0.000147 | 0.000421 |
| CDKN2A | 0.845535 | 15.83917 | 4.227488 | 0.003737 | 0.008428 |
| CDO1 | 2.590983 | 0.663328 | -1.96571 | 1.66E-09 | 3.75E-08 |
| CHAC1 | 0.671191 | 3.115696 | 2.21476 | 6.95E-09 | 1.13E-07 |
| CISD2 | 8.131327 | 12.73481 | 0.647215 | 6.74E-07 | 3.60E-06 |
| CXCL2 | 23.60827 | 5.44147 | -2.11722 | 5.01E-06 | 2.21E-05 |
| DDIT4 | 49.36761 | 109.0869 | 1.143842 | 0.012267 | 0.022636 |
| DRD5 | 0.004467 | 0.031569 | 2.821217 | 0.007803 | 0.016 |
| DUSP1 | 671.7575 | 107.4463 | -2.64433 | 7.04E-12 | 1.43E-09 |
| ENPP2 | 8.256173 | 4.006352 | -1.04318 | 1.11E-06 | 5.39E-06 |
| FANCD2 | 1.145186 | 4.068506 | 1.828917 | 1.37E-10 | 8.72E-09 |
| FLT3 | 0.33205 | 0.118415 | -1.48755 | 3.37E-05 | 0.000114 |
| FTH1 | 190.1178 | 273.53 | 0.524805 | 0.028157 | 0.045006 |
| GABARAPL1 | 28.74186 | 13.41266 | -1.09956 | 1.07E-08 | 1.45E-07 |
| GPT2 | 3.805796 | 8.622031 | 1.179829 | 2.62E-05 | 9.32E-05 |
| HBA1 | 0.29921 | 0.198176 | -0.59438 | 0.030602 | 0.048157 |
| HELLS | 0.750145 | 2.641521 | 1.816128 | 2.04E-10 | 8.72E-09 |
| HIC1 | 3.086134 | 1.22281 | -1.3356 | 3.41E-09 | 6.92E-08 |
| HILPDA | 7.075457 | 26.06677 | 1.881317 | 1.07E-07 | 8.34E-07 |
| HRAS | 23.98106 | 40.85062 | 0.768462 | 0.000844 | 0.002168 |
| HSPA5 | 106.9193 | 203.9505 | 0.931696 | 8.13E-08 | 7.18E-07 |
| IL33 | 17.96555 | 6.157678 | -1.54477 | 7.25E-09 | 1.13E-07 |
| IL6 | 41.79983 | 4.893297 | -3.09462 | 1.44E-08 | 1.75E-07 |
| ISCU | 25.12152 | 17.25974 | -0.54151 | 6.63E-06 | 2.73E-05 |
| JDP2 | 9.168808 | 3.866168 | -1.24583 | 3.54E-08 | 3.99E-07 |
| JUN | 182.3499 | 66.69926 | -1.45097 | 8.28E-09 | 1.20E-07 |
| LINC00336 | 0.005745 | 0.047335 | 3.042467 | 0.001555 | 0.003671 |
| LINC00472 | 0.281223 | 0.066773 | -2.07438 | 4.04E-08 | 4.31E-07 |
| LPIN1 | 3.27298 | 1.872527 | -0.80562 | 0.018081 | 0.030844 |
| LURAP1L | 8.304219 | 5.612882 | -0.5651 | 0.004971 | 0.010511 |
| MAFG | 10.85393 | 6.601108 | -0.71744 | 3.75E-05 | 0.000121 |
| MIOX | 0.022452 | 0.377107 | 4.070034 | 4.52E-08 | 4.58E-07 |
| MT1G | 3.828318 | 10.50269 | 1.455976 | 0.010307 | 0.019555 |
| MUC1 | 19.9035 | 65.26317 | 1.713247 | 0.027614 | 0.044804 |
| MYB | 0.390793 | 1.59929 | 2.032955 | 3.47E-05 | 0.000116 |
| NCOA4 | 55.73588 | 38.51147 | -0.53332 | 0.000327 | 0.00091 |
| NFE2L2 | 42.78501 | 24.36205 | -0.81247 | 1.47E-08 | 1.75E-07 |
| NFS1 | 3.530527 | 5.875981 | 0.734946 | 2.92E-07 | 2.02E-06 |
| NGB | 0.018955 | 0.45907 | 4.598084 | 0.009283 | 0.018296 |
| NOX4 | 0.401883 | 0.582941 | 0.536576 | 0.010072 | 0.019288 |
| NOX5 | 0.06363 | 0.165375 | 1.377962 | 0.017494 | 0.030096 |
| NRAS | 10.43794 | 17.10702 | 0.712751 | 1.50E-05 | 5.52E-05 |
| OTUB1 | 16.21126 | 26.58301 | 0.713508 | 2.99E-07 | 2.02E-06 |
| PCK2 | 6.807965 | 16.27328 | 1.257209 | 7.30E-08 | 6.80E-07 |
| PHKG2 | 3.180917 | 5.809103 | 0.868873 | 7.43E-07 | 3.87E-06 |
| PLIN4 | 33.74325 | 5.161912 | -2.70862 | 6.70E-07 | 3.60E-06 |
| PML | 5.941048 | 9.539833 | 0.683246 | 0.0019 | 0.004433 |
| PRDX1 | 163.9751 | 277.6165 | 0.759617 | 8.92E-07 | 4.42E-06 |
| PRKAA2 | 2.248573 | 0.388617 | -2.53259 | 6.74E-07 | 3.60E-06 |
| PROM2 | 23.81449 | 44.40434 | 0.898861 | 0.008957 | 0.018003 |
| PSAT1 | 6.54968 | 17.9487 | 1.454383 | 5.98E-06 | 2.53E-05 |
| PTGS2 | 59.01404 | 14.79821 | -1.99564 | 1.02E-07 | 8.24E-07 |
| RPL8 | 555.9899 | 1028.106 | 0.886858 | 1.22E-06 | 5.76E-06 |
| RRM2 | 7.288324 | 20.45926 | 1.489095 | 1.43E-07 | 1.08E-06 |
| SCD | 22.39781 | 92.19654 | 2.041355 | 6.42E-07 | 3.60E-06 |
| SIRT1 | 8.18655 | 4.308472 | -0.92608 | 5.76E-07 | 3.44E-06 |
| SLC2A12 | 1.709751 | 0.659074 | -1.37527 | 0.004319 | 0.009426 |
| SLC2A3 | 22.97403 | 8.350926 | -1.46 | 8.16E-07 | 4.14E-06 |
| SLC2A6 | 2.386938 | 7.337353 | 1.620099 | 3.99E-05 | 0.000125 |
| SLC2A8 | 14.36382 | 8.824161 | -0.70291 | 0.010421 | 0.019589 |
| SLC38A1 | 11.21569 | 22.08311 | 0.977425 | 4.13E-06 | 1.86E-05 |
| SLC3A2 | 22.54062 | 43.78444 | 0.957891 | 2.97E-06 | 1.37E-05 |
| SLC40A1 | 20.51227 | 14.03768 | -0.54718 | 0.000121 | 0.000355 |
| SNX4 | 9.415659 | 15.37844 | 0.707775 | 3.51E-07 | 2.30E-06 |
| SQSTM1 | 50.16453 | 34.27411 | -0.54955 | 3.12E-05 | 0.000109 |
| SRC | 20.65352 | 37.40184 | 0.856722 | 0.007708 | 0.015967 |
| STAT3 | 32.51269 | 22.93078 | -0.50372 | 0.000867 | 0.002173 |
| STEAP3 | 6.955353 | 16.51006 | 1.247149 | 1.36E-05 | 5.22E-05 |
| STMN1 | 16.09991 | 58.01474 | 1.849367 | 8.69E-08 | 7.35E-07 |
| TAZ | 4.567859 | 7.172033 | 0.650864 | 0.000138 | 0.0004 |
| TFAP2C | 5.333661 | 10.22188 | 0.938463 | 0.001163 | 0.00281 |
| TFR2 | 0.192823 | 1.202769 | 2.641007 | 2.03E-07 | 1.47E-06 |
| TFRC | 13.32464 | 32.05527 | 1.266465 | 0.000107 | 0.000318 |
| TLR4 | 3.449436 | 2.155561 | -0.6783 | 3.82E-05 | 0.000121 |
| TNFAIP3 | 34.64593 | 12.74774 | -1.44244 | 1.27E-05 | 4.95E-05 |
| TP63 | 11.13168 | 20.94658 | 0.912043 | 0.017346 | 0.030096 |
| TRIB3 | 3.165933 | 10.76683 | 1.765891 | 4.35E-07 | 2.67E-06 |
| TSC22D3 | 56.5829 | 21.38198 | -1.40397 | 4.67E-09 | 8.61E-08 |
| TXNIP | 208.4273 | 145.7216 | -0.51633 | 0.001404 | 0.003353 |
| TXNRD1 | 39.88059 | 19.5494 | -1.02856 | 0.004701 | 0.010152 |
| VDAC2 | 35.10546 | 24.03702 | -0.54644 | 0.000226 | 0.000636 |
| VLDLR | 4.583531 | 1.970522 | -1.21788 | 0.025287 | 0.041397 |
| WIPI2 | 7.608399 | 11.73166 | 0.624743 | 4.14E-07 | 2.63E-06 |
| YWHAE | 91.56195 | 149.4905 | 0.707234 | 6.72E-06 | 2.73E-05 |
| ZEB1 | 17.17626 | 2.158263 | -2.99247 | 7.37E-08 | 6.80E-07 |
| ZFP36 | 863.5709 | 141.293 | -2.61162 | 3.01E-10 | 8.74E-09 |
| ZFP69B | 0.490432 | 1.166984 | 1.250661 | 5.25E-06 | 2.27E-05 |
| ZNF419 | 0.636806 | 1.209149 | 0.925067 | 3.33E-05 | 0.000114 |
