# Supplementary material for: Biological Functions and Prognostic Value of Ferroptosis-Related Genes in Bladder Cancer
Source: Front Mol Biosci. 2021 Nov 17;8:631152. doi: 10.3389/fmolb.2021.631152 (PMC8635965; doi:10.3389/fmolb.2021.631152)
Supplement: Supplementary file 3 [file Table2.DOCX]

Table S2. Detailed information on primers of ferroptosis-related genes

| **Gene** | **Primer-forward** | **Primer-reverse** |
| --- | --- | --- |
| ISCU | GGTCCCTTGACAAGACATCTAA | CTTTCCTTTCACCCATTCAGTG |
| NFE2L2 | GCCCTCACTGGATAAAGAA | CATGCCGTTGCTGGTAC |
| MAFG | GACCCCCAATAAAGGAAACAAG | TTCTCCAGCTCCTCCTTCT |
| ZEB1 | TAAAGTGGCGGTAGATGGTA | ACTGTTTGTAGCGACTGGATT |
| VDAC2 | CTTCAGTAAACCTTGCTTGGAC | GCTAGAGTTGTTGACTTTTGCA |
| TXNIP | GTTCAGAAGATCAGGCCTTCTA | TCCAGGAACGCTAACATAGATC |
| SCD | CTTTCTGATCATTGCCAACACA | TGTTTCTGAAAACTTGTGGTGG |
| JDP2 | AGGCTTCCTTTTGGTTCCCG | GAGAGCACCTCCCTGCAAAC |
